# Iatrogenic Cushing syndrome

**DOI:** 10.1210/jcemcr/luag073

**Published:** 2026-04-24

**Authors:** Prerna Dogra, Michele C Canale

**Affiliations:** Department of Head and Neck-Endocrine Oncology, Moffitt Cancer Center, Tampa, FL 33612, USA; Department of Head and Neck-Endocrine Oncology, Moffitt Cancer Center, Tampa, FL 33612, USA

**Keywords:** exogenous Cushing syndrome, moon facies, large striae, adrenal atrophy, hypothalamic-pituitary-adrenal axis suppression

## Image legend

A 23-year-old woman with common variable immunodeficiency–associated pancytopenia underwent hematopoietic stem cell transplantation from a matched unrelated donor 18 months before presentation. Her post-transplant course was complicated by gastrointestinal and pulmonary graft-vs-host disease (GVHD), for which she received prolonged and repeated courses of high-dose prednisone. Two weeks after tapering and discontinuation of prednisone, laboratory evaluation showed an 8 Am serum cortisol <1 mcg/dL (SI < 27.6 nmol/L) (reference range 3.7-19.4 mcg/dL [SI: 102.1-535.2 nmol/L]) and corticotropin (ACTH) 3.5 pg/mL (SI 0.77 pmol/L) (reference range 7.2-63.3 pg/mL [SI 26.4-232.4 pmol/L]), prompting endocrinology referral.

On examination, she had a round plethoric face (Panel A), dorsocervical fat pad, centripetal obesity, large red abdominal striae (Panel B), multiple ecchymoses, and muscle wasting [1]. Abdominal CT demonstrated bilateral adrenal atrophy (Panel C). A diagnosis of iatrogenic Cushing syndrome and secondary adrenal insufficiency from glucocorticoid therapy induced prolonged hypothalamic-pituitary-adrenal axis suppression was made [1, 2]. She was started on physiologic-dose hydrocortisone replacement [2]. Multidisciplinary consensus is to minimize high-dose glucocorticoids, though intermittent courses remain necessary for GVHD.

**Figure:**
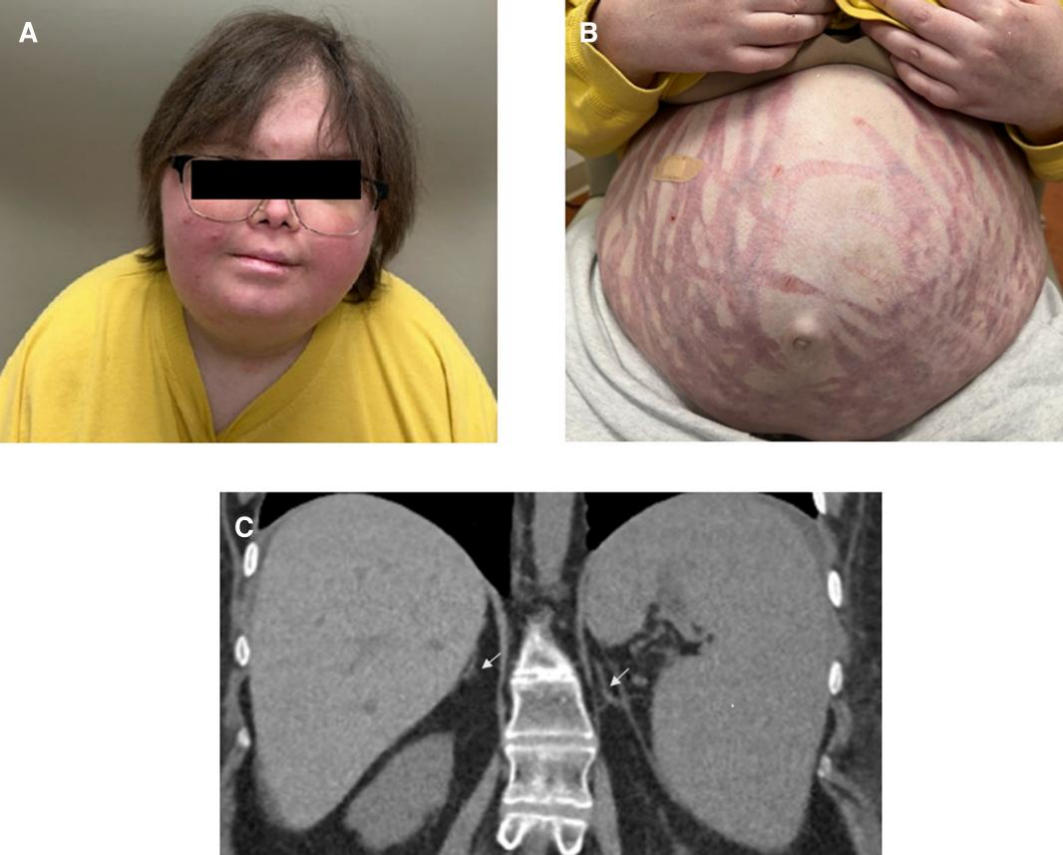


## References

[1] Hopkins RL, Leinung MC. Exogenous Cushing's syndrome and glucocorticoid withdrawal. Endocrinol Metab Clin North Am. 2005;34(2):371‐384. 15850848 10.1016/j.ecl.2005.01.013

[2] Beuschlein F, Else T, Bancos I, et al European Society of Endocrinology and Endocrine Society joint clinical guideline: diagnosis and therapy of glucocorticoid-induced adrenal insufficiency. Eur J Endocrinol. 2024;190(5):G25‐G51. 38714321 10.1093/ejendo/lvae029

